# Placental Hofbauer cells limit HIV-1 replication and potentially offset mother to child transmission (MTCT) by induction of immunoregulatory cytokines

**DOI:** 10.1186/1742-4690-9-101

**Published:** 2012-12-05

**Authors:** Erica L Johnson, Rana Chakraborty

**Affiliations:** 1 Department of Pediatrics and Children’s Healthcare of Atlanta, Emory University, Atlanta, GA, 30322, USA; 2 Division of Infectious Diseases, Emory University School of Medicine, 2015 Uppergate Drive NE, Atlanta, GA, 30322, USA

**Keywords:** HIV-1, Mother to child transmission, Placenta, Hofbauer Cells, Immunoregulation, Cytokines

## Abstract

**Background:**

Despite readily detectable levels of the HIV-1 (co)-receptors CD4, CCR5 and DC-SIGN on placental macrophages (Hofbauer Cells [HCs]), the rate of HIV-1 infection *in utero* in the absence of interventions is only 7% of exposed infants. Here, we examine the replication kinetics of human HCs to the primary isolate HIV-1_BaL_. We also determined the infectivity of HIV-1-exposed HCs by co-culturing with isolated cord and peripheral blood mononuclear cells [CBMCs, PBMCs]. To understand the limiting nature of HCs to HIV-1 replication, we examined the effect of endogenously secreted cytokines on replication kinetics.

**Results:**

HCs have reduced ability to replicate HIV-1 *in vitro* (p < 0.01) and to transmit virus to CBMCs and PBMCs (p < 0.001 for both) compared to standard infections of MDMs. HCs were shown to release HIV-1 particles at levels comparable to MDMs, however exhibit significant decreases in viral transcription (*gag* and *env*), which may account for lower levels of HIV-1 replication. Un-stimulated HCs constitutively express significantly higher levels of regulatory cytokines, IL-10 and TGF-β, compared to MDMs (p < 0.01), which may contribute to immunoregulatory predominance at the placenta and possibly account for down-regulation of HIV-1 replication and infectivity by HCs. We further demonstrate that these regulatory cytokines inhibit HIV-1 replication within HCs *in vitro*.

**Conclusion:**

HCs have reduced ability to replicate and disseminate R5-tropic HIV-1_BaL_*in vitro* and potentially offset mother to child transmission (MTCT) of HIV-1 by the induction of immunoregulatory cytokines. Despite the potential for migration and infectivity, HCs are not present in the neighboring fetal circulation. These results implicate HCs as important mediators of protection at the feto-maternal interface during ongoing HIV-1 exposure.

## Background

In 2008, UNAIDS estimated that 430,000 new HIV-1 infections occurred in children under 15 years of age; most occurred from mother to child transmission (MTCT) during labor and delivery or through breastfeeding [1]. The risk of *in utero* transmission, however, is less than 7%; so that even in the absence of virologic suppression with maternal antiretroviral therapy, over 90% of HIV-1-exposed newborns are “naturally” protected from infection *in utero*. These observations suggest the placenta has evolved mechanisms that restrict establishment of viral infection at the feto-maternal interface. Elucidating these mechanisms may help determine biologic correlates of protection against HIV-1 transmission in humans.

Cytokines influence placental development, fetal growth and immunity, and HIV-1 replication in infected cells [2]. Thus, while strong T_H_1-prototype cytokine responses are associated with recurrent abortions [3] and enhanced HIV-1 replication *in vitro*[4], regulatory cytokine responses have been shown to inhibit HIV-1 replication [5]. Placentas from non-transmitting mothers appear to sustain an immunoregulatory predominance while placentas from transmitting mothers exhibit a pro-inflammatory pattern of cytokine release [5]. It is unknown whether this intrinsic immunoregulatory adaption within the placenta limits HIV-1 replication, thereby offsetting vertical transmission.

The feto-maternal interface is characterized by intimate contact between uterine decidual cells and invading chorionic villi. An individual villus is lined by trophoblasts, which enclose connective tissue stroma containing fetal blood vessels and numerous placental macrophages or Hofbauer cells (HCs). The chorionic villi are directly bathed in maternal blood. An HIV-1 virion can potentially encounter HCs after breaching the trophoblast cell layer. Intriguingly, HCs express the HIV-1 (co)-receptors CD4, CCR5 and CXCR4, and also DC-SIGN on their cell surface [6]. DC-SIGN is a calcium-dependent lectin which binds HIV-1 gp120 with a greater affinity than CD4 [7]. The presence of virus has been detected histologically in HCs [8], which appear to be ideal targets to facilitate HIV-1 transmission. Despite this phenotype the risk of *in utero* transmission remains very low.

Several studies have demonstrated potential modes of HIV-1 transplacental passage [9,10]. Recently, maternal cells were identified in fetal lymph nodes and noted to induce the development of T regulatory cells that suppress fetal anti-maternal immunity [11]. It would seem plausible that the fetus of an HIV-1-infected mother may be exposed to free and cell-associated virus during gestation. The relatively low risk of *in utero* transmission points to innate and adaptive mechanisms that have evolved in humans to restrict lentiviral infection within the placenta.

We investigated the hypothesis that placental macrophages (HCs) limit HIV-1 replication compared to MDMs through the production and response to regulatory cytokines. Our group and others have reported that HCs exhibit reduced ability to replicate HIV-1 *in vitro*[12,13]. This restriction of HIV-1 replication may occur at the level of viral transcription. To further examine the limiting nature of HCs to viral replication, we examined the phenotype and cytokine profile of these placental macrophages. We noted that cytokines expressed by HCs have inhibitory effects on HIV-1 replication *in vitro* and that despite the potential for migration and infectivity, HCs are not present in the neighboring fetal circulation. These results implicate HCs as important mediators of protection at the feto-maternal interface during HIV-1 exposure.

## Results

### HCs are potential targets for HIV-1 infection

**Figure 1:**
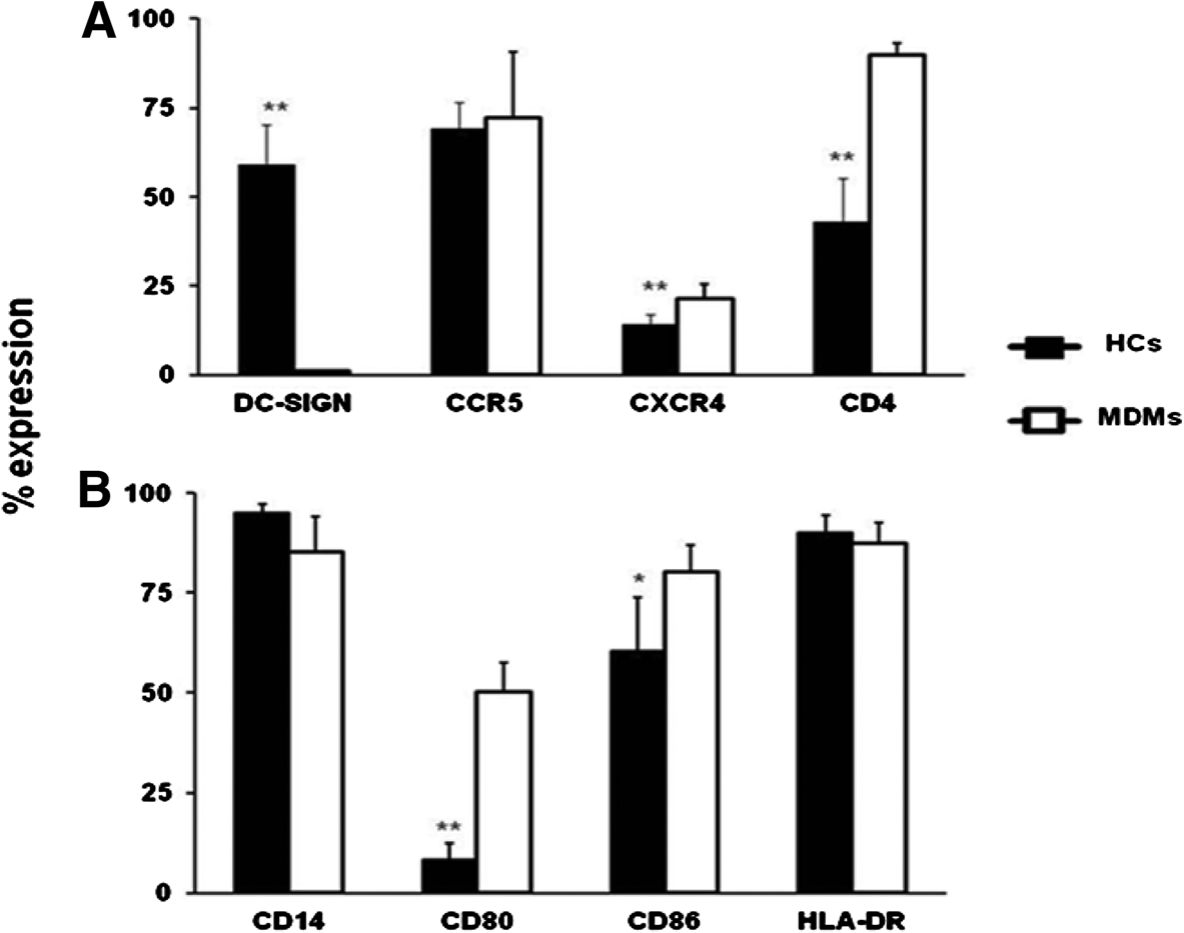
**HCs are potential targets for HIV-1 infection but express low levels of the co-stimulatory signal CD80.** The cell surface expression of molecules involved in (**A**) HIV-1 entry and (**B**) activation were analyzed on HCs and MDMs. Fluorescence intensity was measured in cells incubated with antibodies against the pattern recognition receptor (CD14), the co-stimulatory markers (CD80-PE and CD86-APC), MHC II marker (HLA-DR-PE) and HIV-1 receptors (CD4-APC, CCR5-PerCp-Cy5.5, CXCR4-PE-Cy7, and DC-SIGN-PE). (**A**) Phenotypic markers are represented as a percentage of expression in purified populations of HCs and MDMs. Results are presented as a mean percentage (±SE) of the total population, which are positive for the marker. Data shown are representative of three independent experiments from different donors (* *p* < *0*.*05*, **p < *0*.*01*).

We analyzed the cell surface expression of molecules involved in HIV-1 entry and activation on HCs isolated from term placentas. CD14+ HCs express the HIV-1 co-receptors CD4, DC-SIGN and CCR5, and to a lesser extent CXCR4 (Figure 1A). Compared to monocyte-derived macrophages (MDMs), HCs express similar levels of CCR5 and CXCR4. The percentage fluorescence expression of CD4 on HCs was less than in MDMs, and detectable concentrations of DC-SIGN were only noted on the cell surface of HCs. T-cell activation requires co-stimulatory signals through binding of the CD28 receptor with cognate ligands, CD80/CD86, located on the surface of antigen presenting cells (APCs). When co-stimulation is coupled with a signal through the T-cell receptor (TCR), T-cell proliferation and cytokine secretion are induced. HCs and MDMs expressed comparable levels of MHC class II cell surface receptor, HLA-DR; however, lower levels of CD80 were expressed on HCs, while CD86 was similar to that expressed by MDMs (Figure 1B). These results may suggest that HCs possess the ability to engage the TCR, but without sufficient CD80 stimulation are unable to induce a robust T-cell response.

### HCs have reduced ability to replicate HIV-1 compared to MDMs

**Figure 2:**
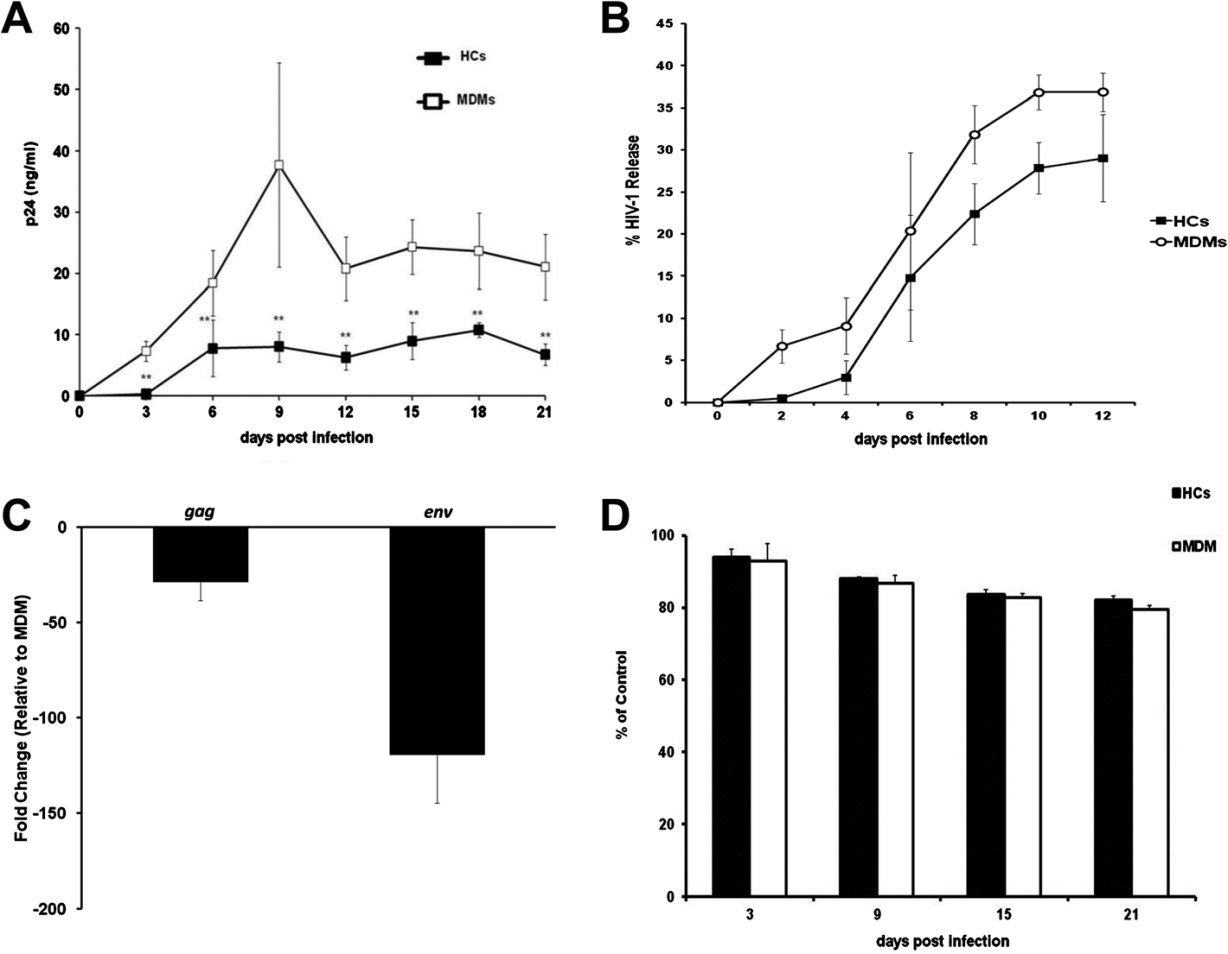
**HIV-1 replication in HCs and MDMs.** Since HCs appear to be primary targets for HIV-1 infection, we compared HIV-1 replication in HCs to MDMs. HCs (closed square) and MDMs (open square) infected by HIV-1_BaL_*in vitro* showed differences in HIV-replication over time. HIV-1 replication was measured in the cell supernatants by HIV-1 p24 viral antigen ELISA (**A**). HIV-1 release over time from HCs and MDMs was quantified as the percentage of p24 antigen in supernatants as a fraction of p24 antigen in supernatant plus cell lysate (**B**). Six days post infection, mRNA levels were measured by real-time PCR to determine fold-changes in *env* and *gag* transcription (**C**). Cell cytotoxicity was assessed by MTT assay (**D**). The ratio of optical density from infected cells to optical density from control cells reflected the percentage of surviving cells. Data shown are expressed as the mean ± SE of triplicate samples and are representative of 3 independent experiments from 10 different donors (**p < *0*.*01*).

Since HCs are susceptible to HIV-1 infection, studies were conducted to determine the potential permissiveness of decidual HCs to experimental *in vitro* infection using cell-free HIV-1. We compared HIV-1 replication in HCs to MDMs using R5-tropic HIV-1_BaL_ at a TCID_50_ of 0.2 per cell. HCs were productively infected, as observed by a detectable increase in the levels of p24 in the supernatant fluid over time, ranging from 0–10 ng/ml (Figure 2A). Mean p24 antigen production in HCs was significantly less than that detected in MDMs cultured in parallel at the same TCID_50_ (p < 0.01). The exact magnitude of restriction varied slightly from donor to donor, but the reduced ability of HCs to replicate HIV-1 *in vitro* was consistent for each donor [(N =16) (see Additional files 1 and 2). To verify HCs are productively infected with HIV-1, we utilized electron microscopy (EM) studies to demonstrate viral assembly within intracellular compartments and at the plasma membrane surface (see Additional file 3). To examine HIV-1 assembly and release in HCs, we compared particle release efficiency between the HCs and MDMs. Levels of HIV-1 release are expressed as a percentage of p24 antigen in supernatants as a fraction of p24 antigen in supernatant plus cell lysate. No significant differences were found in particle release between the two cell types (Figure 2B). These results indicate restriction does not occur at the point of particle assembly/release. To further account for the reduced ability of HCs to replicate HIV-1, we monitored viral gene transcription 6 days post infection using real-time PCR. We found that HIV-1-infected HCs showed a 28-fold decrease in *gag* mRNA (average Ct values) and a 119-fold decrease in *env* mRNA compared to HIV-1-infected MDMs (Figure 2C). These results demonstrate that HCs have decrease levels of viral transcription, which may account for lower levels of HIV-1 replication. To rule out any possible effect on cell viability that might interfere with the evaluation of HIV-1 replication, a colorometric MTT assay was performed to measure the cell viability. Based on this assay, no significant difference in cell viability was observed on comparing uninfected and HIV-1-infected MDMs and HCs; therefore, the effect is not a consequence of reduced cell viability (Figure 2D). These results demonstrate that placental HCs are permissive to HIV-1_BaL_ but replicate virus to a significantly lesser extent than MDMs. The findings are consistent with observations on decidual CD14+ cells and placental tissue obtained from the first trimester of pregnancy which were also permissive to R5 HIV-1 infection but to a lesser degree than peripheral blood macrophages [14].

### HIV-1-infected HCs have limited capacity to transmit virus to cord blood mononuclear cells (CBMCs)

**Figure 3:**
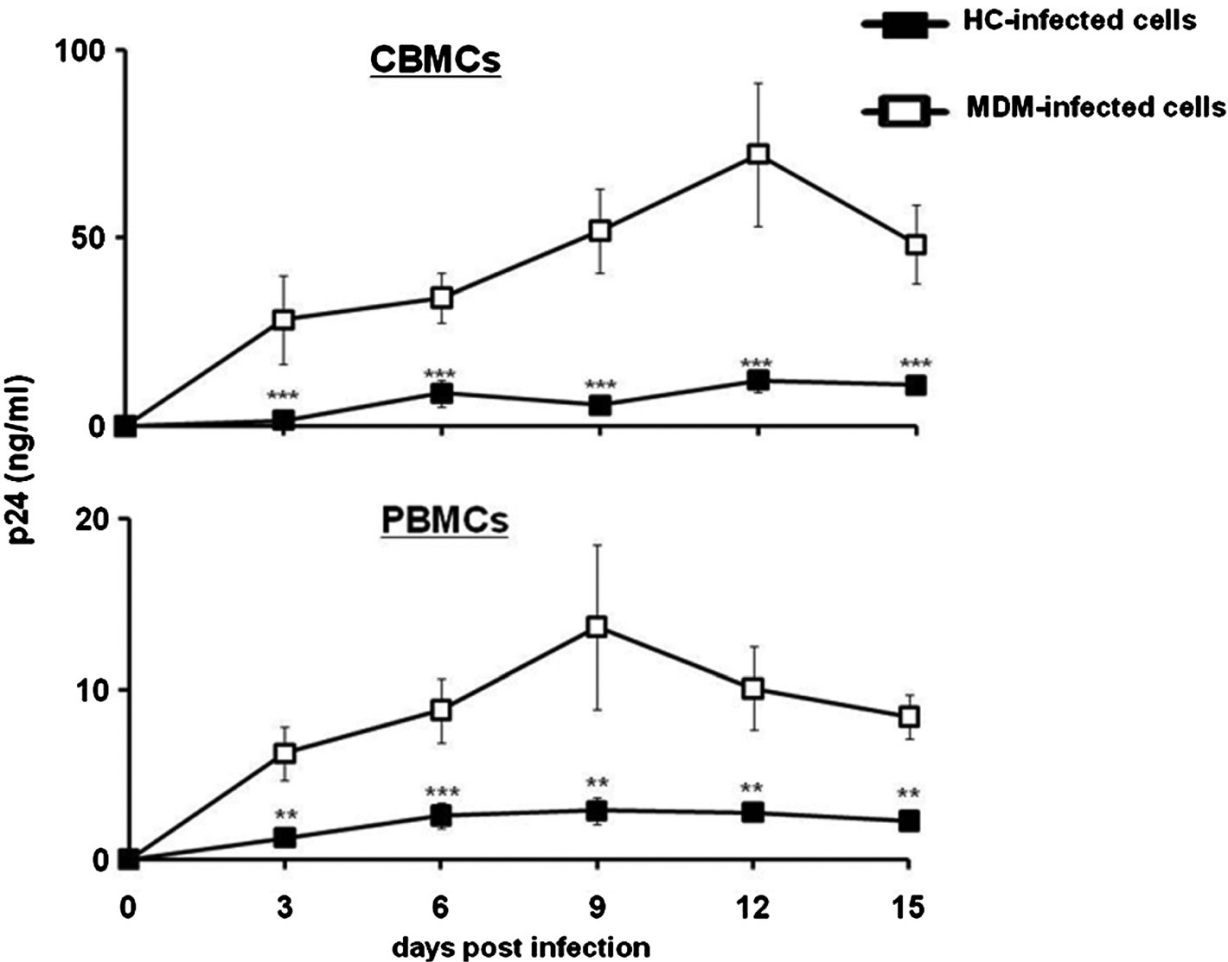
**HIV-1-infected HCs have the capacity to infect CBMCs and PBMCs.** In order to determine the infectivity of HCs, HIV-1_BaL_ infected HCs and MDMs were co-cultivated separately with (**A**) CBMCs and (**B**) PBMCs. After 6 h, the CBMCs and PBMCs were cultured separately, and HIV-1 replication was measured in the cell supernatants by HIV-1 p24 viral antigen ELISA. Closed squares represent HC-infected cells and open squares indicate MDM-infected cells. Data shown are expressed as the mean ± SE of triplicate samples and are representative of 3 independent experiments from different donors (**p < *0*.*01*; ***p < *0*.*001*).

HIV-1_BaL_-infected HCs (or MDMs) were co-cultured with PHA-activated CBMCs. After 6 hours of culture, non-adherent CBMCs were collected and cultured separately from HCs. CBMCs co-cultured with HIV-1-infected HCs had significantly lower concentrations of p24 than CBMCs co-cultured with HIV-1-infected MDM’s (Figure 3A). A similar trend was also noted with PBMCs (Figure 3B). HIV-1-infected HCs can productively transmit virus to neighboring CBMCs, however their relative capacity to disseminate virus is significantly less than MDMs.

### Placental HCs may offset MTCT by inducing regulatory cytokines

**Figure 4:**
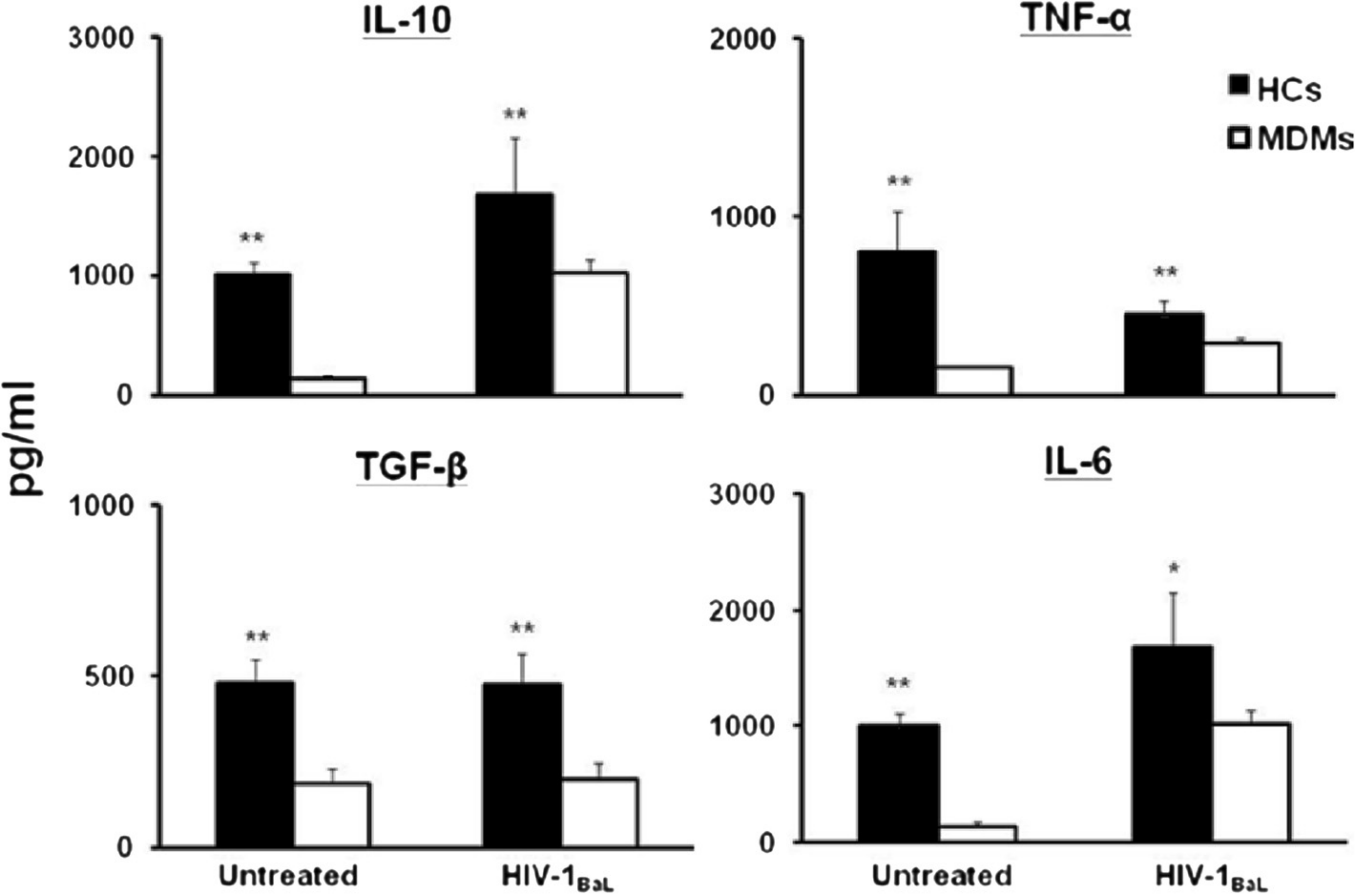
**Placental HCs may offset MTCT by inducing T**_**H**_**2 cytokine bias.** HCs and MDMs were either infected with HIV-1_BaL_, or left uninfected for 48 h. Supernatants were collected and measured for production of IL-10, TGF-β, IL-6, and TNF-α. Cytokines were measured using a Quantikine ELISA assay. Data shown are expressed as the mean ± SE of triplicate samples and are representative of 3 independent experiments from different donors (* *p* < *0*.*05*, **p < *0*.*01*).

Since cytokines have been noted to influence the susceptibility of hematopoietic cells to HIV-1 infection, the supernatant of HCs, and for comparison MDMs, were assayed for a battery of cytokines. Purified HCs and MDMs were cultured with media alone or media containing R5-tropic HIV-1_BaL_ for 48 h and the supernatant analyzed for the concentrations of IL-4, IL-6, IL-10, TGF-β1, TNF-α and IFN-γ. Similar to previously described regulatory macrophages [15], supernatant from HCs constitutively expressed significantly higher concentrations of the immunosuppressive cytokines IL-10 and TGF-β1 compared to un-stimulated MDMs (Figure 4). IL-10 expression increased significantly in HIV-1-infected HCs compared to uninfected cells, while TGF-β expression remained unchanged. The T_H_1 cytokine TNF-α was significantly up-regulated in HCs at baseline, while HIV-1 infection caused a reduction in secreted TNF-α comparable to infected-MDMs (Figure 4). Thus HCs intrinsically exhibit immunosuppressive cytokine predominance similar to the regulatory phenotype noted at the feto-maternal interface [11]; this adaptation is observed following HIV-1 infection of HCs *in vitro*.

### Exogenous regulatory cytokines, normally expressed by the placenta, inhibit HIV-1 replication in HCs *in vitro*

**Figure 5:**
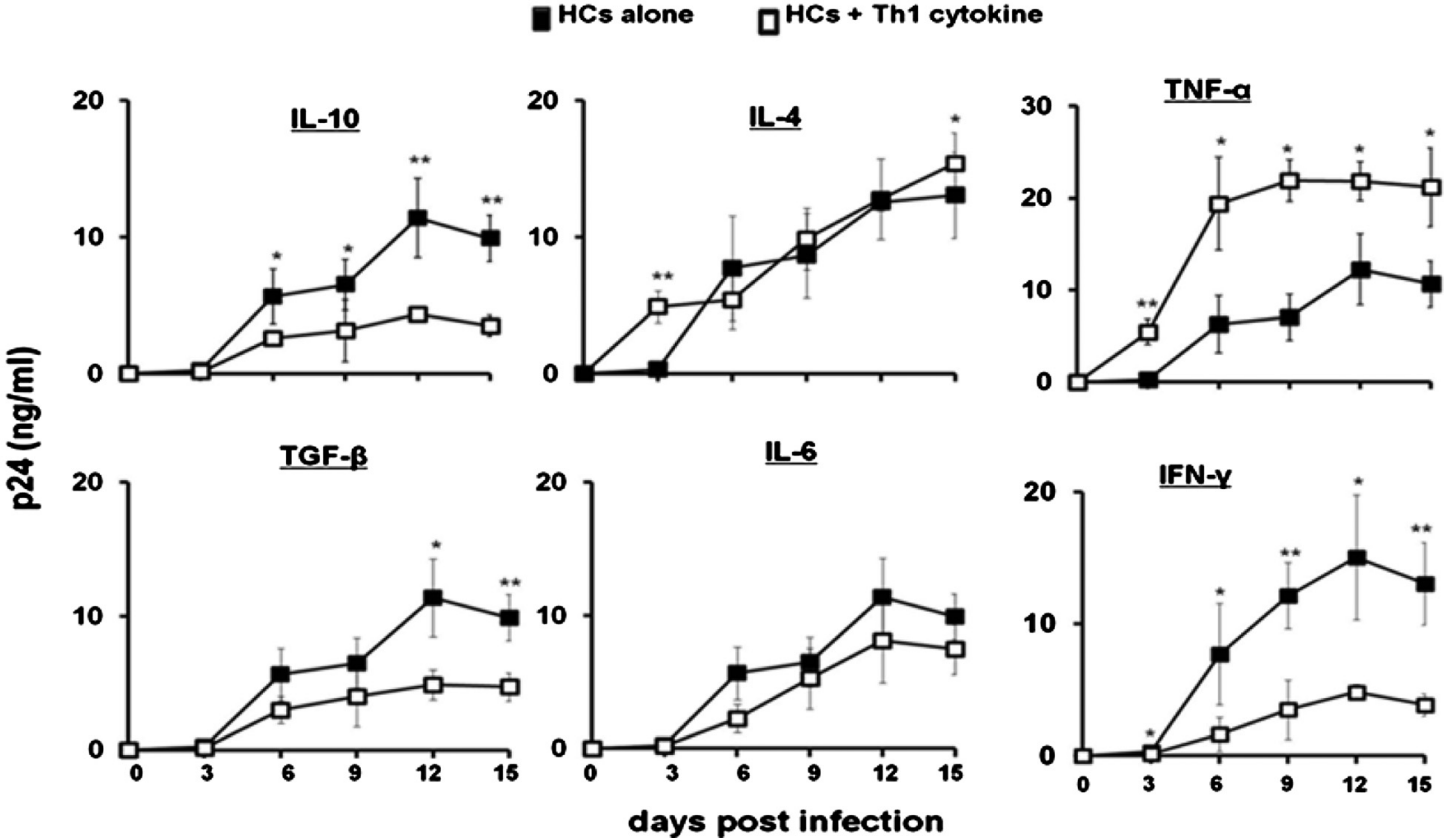
**Exogenous T**_**H**_**2-type cytokines, IL-10 and TGF-β, have inhibitory effects on HIV-1 replication in HCs.** HCs were left untreated or pretreated for with IL-10, TGF-β, TNF-α, IFN-γ, IL-4 and IL-6 prior to infection with HIV-1_BaL_. HIV-1 replication was measured in the cell supernatants by HIV-1 p24 viral antigen ELISA. Data shown are expressed as the mean ± SE of triplicate samples and are representative of 3 independent experiments from different donors (* *p* < *0*.*05*, **p < *0*.*01*).

HCs were pretreated with or without IL-10, TGF-β, IL-4, IL-6, IFN-γ, and TNF-α prior to infection with HIV-1_BaL_. Regulatory cytokines, IL-10 and TGF-β, significantly lowered HIV-1 replication by HCs over time (Figure 5). Exogenous administration of IL-4 did not influence, while IL-6 induced a slight decrease in HIV-1 replication. Interestingly, the prototypic T_H_1 cytokine, IFN-γ, stimulated a drastic reduction in HIV-1 replication in HCs. However, mean p24 antigen production was significantly higher in HIV-1-infected HCs treated with TNF-α. Numerous studies have demonstrated the dichotomous effects of cytokines on HIV-1 replication by MDMs. Generally, inflammatory cytokines significantly up-regulate HIV-1 replication in human macrophages [16], while immunoregulatory cytokines have the converse effect by suppressing viral replication in these cells [17]. These data suggest that differential cytokine signaling at the feto-maternal interface may provide an environment that limits HIV-1 replication within HCs upon infection.

### HCs migrate at similar rates to MDMs after stimulation by CCR5 ligands *in vitro* but are not detected in the cord blood

**Figure 6:**
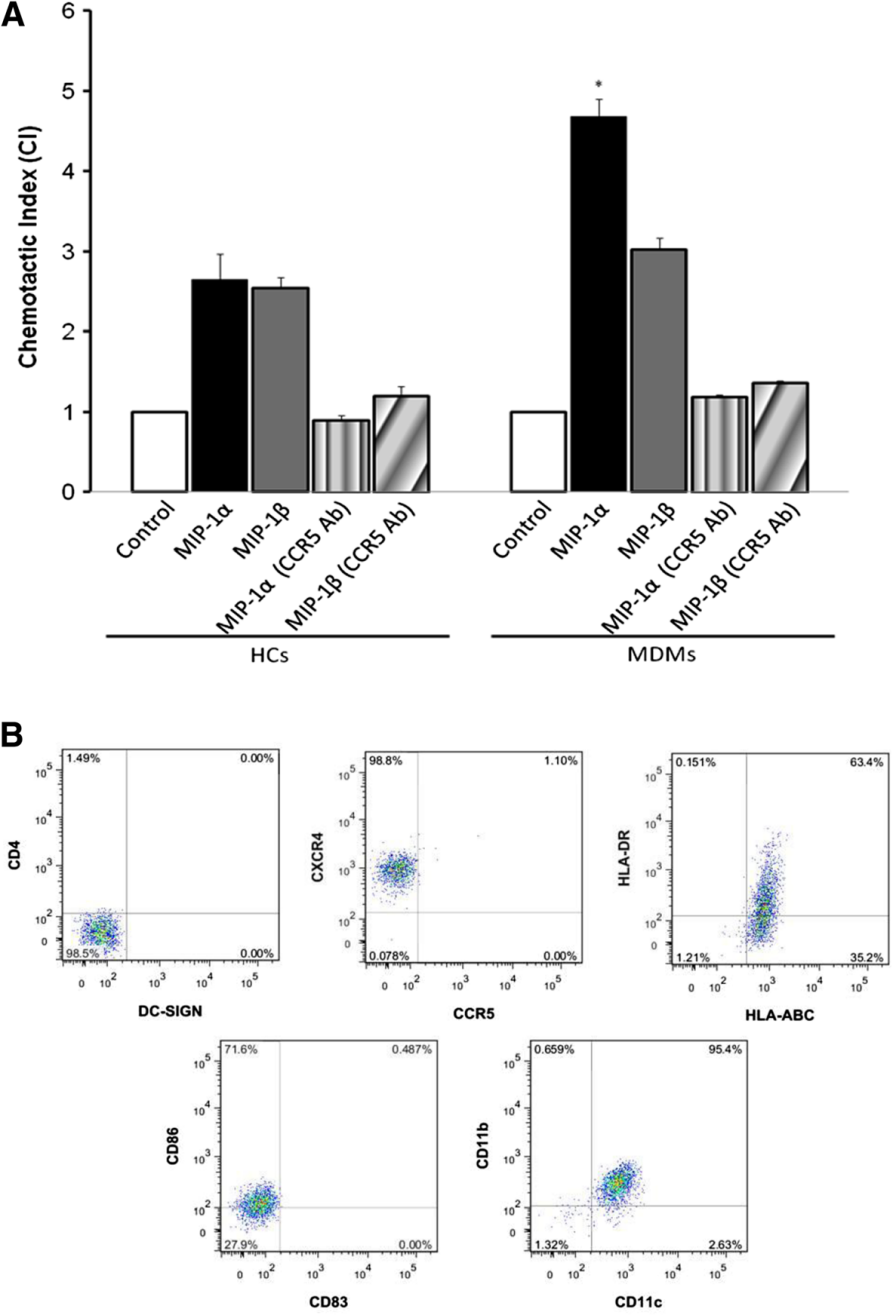
**MIP-1α and MIP-1β induce CCR5-mediated chemotaxis of HCs*in vitro*but are not detected in the cord blood.** MIP-1α and MIP-1β ligands for the CCR5 receptor play an important role in modulating immune responses. To examine CCR5-induced migration (**A**), HCs and MDMs were pretreated with or without human CCR5 monoclonal antibody (5 μg/ml), placed in Transwell inserts, and stimulated with MIP-1α (10 ng/ml) and MIP-1β (10 ng/ml). Results are expressed as chemotactic index (CI). CI is calculated by dividing the mean number of cells migrating toward chemoattractants by that toward control medium. Data shown are means ± SE of three independent experiments from different donors (* *p* < *0*.*05*). To detect the presence of HCs in cord blood (**B**), cells were stained with PE-DC-SIGN, PE-Cy7-CD4, PerCP-CCR5, PE-Cy7-CXCR4, PE-HLA-ABC, APC-HLA-DR, APC-CD11b, PE-CD11c, PE-83, APC-CD86, and FITC-CD14. Quadrants were set according to uninfected control staining. Cells were gated on CD14^+^ cells. Flow cytometry pictures show a representative of 5 different donors.

HCs and MDMs express CCR5, which typically facilitates their mobilization towards MIP-1α and MIP-1β upon activation. In order to examine HC-CCR5-induced migration, *in vitro* assays were used to quantify the number of cells that migrated in response to control media, and media conditioned with MIP-1α and MIP-1β. Migration in HCs and MDMs towards MIP-1α and MIP-1β was CCR5-mediated, and was abrogated by the addition of specific CCR5 antibody. While HCs and MDMs migrated at comparable rates in response to MIP-1β, HCs migrated towards MIP-1α less vigorously than MDMs in repeated experiments (Figure 6A). Therefore, HCs do possess the ability to migrate in a CCR5-dependent manner similar to macrophages. However, HCs were not detected in cord blood by flow cytometry (Figure 6B). These findings suggest that HCs lack the chemotactic response to migrate to cord blood, despite their abundance within the chorionic villi, association with neighboring umbilical vessels, and ability to migrate *in vitro* after stimulation with CCR5 ligands.

## Discussion

The placenta represents a unique immunologic site populated with specialized APCs, specifically HCs. Fetal tolerance involves the interplay of immunologic cells within this interface [18-20]; however, their role in HIV-1 transmission is poorly defined. Here, we report that HCs have reduced ability to replicate and disseminate R5-tropic HIV-1_BaL_*in vitro* and potentially offset MTCT of HIV-1 by the induction of immunoregulatory cytokines. In order for induction of antigen-specific tolerance, there is on-going bi-directional trafficking of immune cells *in utero* between mother and fetus [11]. This continuous two-way transfer causes maternal immune cells and free virus (during HIV-1 infection) to interact directly with HCs. We demonstrate that HCs co-express CD4, CCR5 and CXCR4, and that these cells are susceptible *in vitro* to HIV-1_BaL_ infection_._ Similar to DCs, but unlike peripheral macrophages, HCs express very high levels of DC-SIGN [21]. During pregnancy, there is increased expression of DC-SIGN on HCs; this expression has been correlated with increased rates of HIV-1 vertical transmission [22]. Intuitively, the presence of DC-SIGN and HIV-1 (co)-receptors on HCs, could promote viral entry by free or cell-mediated transmission of HIV-1 facilitating infection in the unborn fetus. Despite this phenotype, MTCT events are rare *in utero* which suggest that HCs may be key mediators of protection during HIV-1 exposure. T-cell activation involves specific binding of the T-cell receptor to the antigen/MHC class II complex and requires a second signal mediated by the simultaneous binding of co-stimulatory molecules CD80 and CD86 on APCs. HCs appear to have the ability to engage the T-cell receptor, but without sufficient CD80 co-stimulation, may be unable to induce an immunologic response characterized by T-cell activation. Our data are supported by observations that HCs inhibit T-cell responses in mixed lymphocyte reactions compared to peripheral blood monocytes [23], and induce T-cell anergy in the placenta [24]. Reports also suggest that expression of CD80 and CD86 on APCs is regulated by cytokines like IL-10 [25].

HCs support infection and replication *in vitro* with HIV-1_BaL_ but mean p24 antigen production is significantly less than in peripheral blood MDMs. These data reflect observations in decidual CD14+ cells and placental tissue obtained from the first trimester of pregnancy that were permissive to R5 HIV-1 infection but to a lesser extent than peripheral blood macrophages [5]. Recent findings demonstrate that HCs and MDMs infected with HIV-1_BaL_ have the same concentration of integrated provirus [26], which suggests restriction in viral replication is not a reflection of decreased HIV-1 entry; instead reduced HIV-1 replication may involve intrinsic blocks unique to these cells [27]. Here, we demonstrate that HCs assemble and release virus at rates comparable to MDMs; however, the overall transcription of viral genes by HCs is significantly reduced. These results suggest HCs express or produce intrinsic factors, which may limit viral transcription following infection, thereby offsetting MTCT. Contact-mediated transfer of HIV-1 is an efficient mode of viral dissemination [28]. Since HCs can be productively infected with HIV-1, we examined whether HIV-1 can be transmitted through cell-to-cell contact from infected HCs to PHA-stimulated autologous CBMCs or allogenic PBLs. Given the close proximity of fetal circulating blood with HCs at the placental interface, cell-mediated transmission of HIV-1 may be a plausible mechanism of viral dissemination following primary infection of HCs. We report that HIV-1-infected HCs have a limited capacity to transmit virus to neighboring CBMCs compared to MDMs.

Recently, regulatory macrophages have been characterized as a broad category of macrophages whose primary role is to dampen the inflammatory immune response. These regulatory macrophages produce high concentrations of immunoregulatory cytokines (i.e. IL-10 and TGF-β) and are potent inhibitors of inflammation, despite their ability to secrete many Th1-type cytokines [15]. IL-10, specifically, is a prototypic regulatory cytokine involved in the deactivation of macrophages [29]. Our group and others have shown that IL-10 is produced constitutively by HCs at significantly higher concentrations than in MDMs [30,31]. In addition, we demonstrate that IL-10 inhibits HIV-1 replication in HCs and propose that the high basal production of IL-10 in HCs, similar to regulatory macrophages, plays a central role in limiting their ability to replicate HIV-1 after initial infection, which may offset vertical transmission. Our data also demonstrate that HCs constitutively secrete high concentrations of TGF-β. Studies have shown that TGF-β and IL-10 drive the differentiation and activation of CD4 + CD25^high^FoxP3+ T regulatory cells [32-34]. T regulatory cells are present in the decidua [35], and a reduction of these cells during pregnancy is associated with spontaneous abortions [36]. In addition, we demonstrate that exogenous TGF-β restricts HIV-1 replication in HCs. Interestingly; other investigators have noted that TGF-β may inhibit MTCT of HIV-1 in breast milk [37]. We propose that HCs may function as regulatory macrophages in the placenta through the induction of immunoregulatory cytokines, which limit HIV-1 replication and induce T regulatory cells within the placenta.

TNF-α had opposite effects on HIV-1 replication in HCs. We have shown that HCs produce elevated concentrations of TNF-α compared to MDMs. TNF-α has a pivotal role during parturition [38]. Over-production of TNF-α may cause damage to the placental barrier, allowing transfer of HIV-1 [39] and induction of viral replication [40]. Our data show that exogenous TNF-α significantly up-regulated HIV-1 replication in HCs *in vitro* which may account for why most vertical transmission events occur during parturition. The up-regulation of a pro-inflammatory cytokine at the feto-maternal interface and by HCs is not incongruous with this regulatory phenotype. Instead, this observation emphasizes the importance of maintaining an appropriate spatial, temporal, and quantitative balance of cytokines within the placental milieu.

The β-chemokines, MIP-1α and MIP-1β are ligands for CCR5 and induce migration through the chemokine receptor CCR5, which is highly expressed on HCs and MDMs. Previous time-lapse observation studies indicate that HCs can migrate rapidly over the surface of adherent fibroblastic cells [41]. Here, we show that HCs also have the potential to migrate in a CCR5-mediated manner in response to the β-chemokines. Despite the potential of HC migration in response to CCR5 ligands, this cell type was not detected in cord blood by flow cytometry. This data may reflect observations that show chemotaxis is deficient in neonates, reflecting reduced concentrations of β-chemokines in cord blood [42].

## Conclusion

We demonstrate the restricted ability of HIV-1 to replicate in HCs, which may offset vertical transmission of HIV-1 through an induction of immunoregulatory cytokines. Cytokine levels fluctuate during a normal pregnancy [43]; our data suggest HCs are important mediators to limit inflammatory cytokine responses within the feto-maternal interface to favor regulatory cytokine predominance. A disruption to this balance may facilitate MTCT of HIV-1.

## Methods

### Study subjects

With written informed consent, term (>37 weeks gestation) placentas from 40 HIV-1 and Hepatits B seronegative women were obtained immediately following elective caesarian section without labor from Grady Memorial and Emory Midtown Hospitals in Atlanta, GA. Approval of the study was granted from Emory University Institutional Review Board and Grady Research Oversight Committee.

### Isolation and Culture of HCs and MDMs

In order to isolate HCs, the decidua basalis was dissected from the placenta. The tissue was thoroughly washed in Hank’s balanced salt solution (HBSS) to minimize peripheral blood contamination and mechanically dispersed in complete medium (RPMI supplemented with 10% FBS, 1 mM L-glutamine, and 1% pen/strep). The minced tissue was re-suspended in complete medium containing 1 mg/ml collagenase IV [Sigma Chemical Co., St. Louis, MO], 10 U/ml dispase [Worthington Biochemical Corp., Lakewood, NJ], and 0.2 mg/ml of DNAse I [Sigma] and incubated in a shaking water bath at 37°C for 1 hour. The digested tissue was washed with PBS and passed through a 70 μm cell strainer [BD Falcon, USA]. The mononuclear cell population was isolated by density gradient centrifugation on Histopaque-1077 [Sigma]. CD14^+^ Magnetic Cell Sorting was performed using anti-CD14 magnetic beads [Miltenyi Biotech, Germany] as recommended by the manufacturer. In order to isolate MDMs, PBMCs were obtained from the buffy coats of healthy HIV-1 seronegative blood donors by density gradient centrifugation. Isolated cells were washed, re-suspended in RPMI containing pen/strep (1%), glutamine (1%), and heat-inactivated normal human serum (10%) [Mediatech, VA], and seeded into 75-cm^2^ flasks [Corning Inc., New York]. Non-adherent cells were removed after 2 h of incubation at 37°C. After 24 hours of culture, adherent cells were washed, detached from the flask and counted. Adherent HCs and MDMs were washed with cold EDTA/PBS and detached by incubation with cold EDTA/PBS (2 mM) for 30 min at 4°C. The cells were seeded into 24-well plates and were cultivated for 7 additional days to promote full differentiation into MDMs.

### Flow cytometric analysis of cell surface determinants

Cells were labeled with anti-CD14 (F), -CD80 (PE), -CD83 (PE), -CD86 (APC), -HLA-DR (PE), -CCR5 (PerCp-Cy5.5), -CXCR4 (PE-Cy5), -CD4 (APC), and –DC-SIGN (PE) [BD Biosciences, CA]. Briefly, Fc-blocked HCs were incubated with the conjugate antibodies, and unbound antibody washed from the cells. After the final washes, cells were fixed in 1% paraformaldehyde, and cell surface expression was determined by FACS analysis with FACSCanto [Becton Dickinson, CA], and the results were analyzed with the FlowJo software version 8.4.3 [Tree Star, OR].

### Infection of HCs and MDMs with HIV-1_BaL_

The macrophage-tropic isolate, HIV-1_BaL_ was a gift from Jason Hammonds PhD of Emory University School of Medicine. Macrophages were infected at 0.2 TCID50/cell for 4 hours at 37°C. Cells were washed and fresh media added to the cultures. To monitor viral production and particle release, cell supernatants and lysates were collected at days 0, 2, 3, 4, 6, 8, 9 10,, 12, 15, 18 and 21 post-infection. Viral replication and particle release was detected by p24 released into the supernatant and cell-associated p24 by ELISA [Advanced BioScience Laboratories, Inc., MD].

### Real-time PCR

HCs and MDMs were infected at 0.2 TCID50/cell for 4 hours at 37°C. Cells were washed, and fresh media added to the cultures. After 6 days, cells were harvested and mRNA was extracted using the RNAeasy kit (Qiagen, Valencia, CA). The cDNA was transcribed using QuantiTect RT kit (Qiagen). The primer sequences were as follows: forward primer *env* (5′-GGGGACCAGGGAGAGCATT-3′) and reverse primer *env* (5′-TGGGTCCCCTCCTGAGGA-3′); forward primer *gag* (5′- ACATCAAGCAGCCATGCAAAT-3′) and reverse primer *gag* (5′- ATCTGGCCTGGTGCAATAGG-3′). Real-time PCR was performed using SYBR Green (Qiagen). All reactions were run in triplicate using the Applied Biosystems Prism 7500 Sequence Detection System. Delta-Ct values from the calibrator and experimental groups were measured by subtracting Ct values from target versus the housekeeping transcript, 18S. Each sample is expressed as fold-change relative to HIV-1 infected MDMs.

### MTT assay

Cell cytotoxicity was assessed by 3-(4,5-dimethylthiazol-2-yl)-2,5-diphenyl tetrazolium bromide (MTT, Sigma) mitochondrial dehydrogenase assay. Cells were incubated with a 1: 10 dilution of MTT solution to cell media for 20 min at 37 °C. The extent of MTT conversion to formazan by mitochondrial dehydrogenase was determined by measuring optical density at 490 nm using a microplate reader (Molecular Devices, Sunnyvale, CA). The ratio of optical density from treated cells to optical density from control cells reflected the percentage of surviving cells.

### Culture of human CBMCs and PBMCs

Cord blood samples were collected from umbilical cords of full-term newborns born to HIV-1 seronegative mothers. PBMCs were obtained from healthy, HIV-1 seronegative adults. CBMCs and PBMCs were separated from heparinized whole-blood samples by density gradient centrifugation on Ficoll-Hypaque [Sigma]. The isolated mononuclear cells were washed in sterile PBS and once in RPMI 1640, maintained in complete medium. CBMCs and PBMCs were stimulated with PHA (5 μg/ml) [Sigma] in the basic medium described above for 24–48 hours prior to infection. Stimulated cells were challenged with HIV-1 immediately following PHA stimulation. Following HIV-1 infection, the PHA-stimulated cells were maintained in complete medium supplemented with IL-2 (100U/ml) [R&D Systems, Minneapolis, MN].

### Co-culture assays

Adherent, HIV-1_BaL_ infected HCs and MDMs were co-cultured with CBMCs or PBMCs for 6 hours. Non-adherent cells were gently removed and washed with PBS, counted and plated in complete medium supplemented with IL-2. Viral replication was detected by p24 release into the supernatant by ELISA.

### Cell culture and cytokine measurement

Cells (0.5 x10^6^) were cultured with HIV-1_BaL_ or alone for 2 days. IL-4, IL-6, IL-10, TNF-α, TGF-β, and IFN-γ concentrations were measured by Quantikine ELISA kit [R&D Systems] according to manufacturer’s instructions.

### Cytokine treatment and HIV-1 replication

In order to analyze if exogenous IL-4, IL-6, IL-10, TNF-α, TGF-β, and IFN-γ influenced HIV-1 replication, HCs were pretreated for two hours with cytokines [R&D Systems] then inoculated as described earlier with HIV-1_BaL_. To monitor viral production, cell supernatants were collected at days 0, 3, 6, 9, 12, 15, 18 and 21 post-infection.

### Migration assays

HCs and MDMs were re-suspended in RPMI containing 0.1% BSA and 2 x 10^5^ cells, incubated with human CCR5 monoclonal antibodies (5 μg/ml) or along for 15 min – 2 hours, and added to 24-2well Transwell inserts with 8 μm pore polycarbonate membranes (Corning). Inserts were placed in wells containing 600 μl media plus chemoattractants and incubated at 37°C for 18 hours. Cells that migrated through the membrane were stained and counted microscopically in five random high-powered fields. Chemotactic responses are expressed as CI, calculated by dividing the mean number of cells migrating toward chemoattractants by that toward control medium. MIP-1α and MIP-1β were used for chemotaxis at 10 ng/ml (Peprotech, Rocky Hill, NJ).

### Electron microscopy

HIV-1-infected HCs were fixed with 2.5% glutaraldehyde in 0.1 M cacodylate buffer (pH 7.4) overnight at 4 degree C, and then washed with the same buffer. The cells were then post fixed with 1% osmium tetroxide and 1.5% potassium ferrocyanide in 0.1 M cacodylate buffer for one hour. The cells were subsequently rinsed with de-ionized water, dehydration through an ethanol series ending with absolute ethanol, and then embedded in Eponate 12 resin. Ultrathin sections were cut on a RMC PowerTome XL ultramicrotome at 70 nm, stained with 5% aqueous uranyl acetate and 2% lead citrate, and examined on a JEOL IEM-1400 transmission electron microscope equipped with Gatan UltraScan US1000.894 and Orius SC1000.832 CCD cameras.

### Statistical analyses

Results were analyzed by a one-way analysis of variance (ANOVA). Parametric data were analyzed by using Student’s *t* test (two-tailed) in order to assess whether the means of two normally distributed groups differed significantly. All non-parametric data was analyzed using Mann–Whitney *U* Test. All error bars represent the SE.

## Competing interests

The authors declare that they have no competing interests.

## Authors’ contributions

ELJ: experimental design, performed experiments, data analysis and manuscript preparation; RC: principal investigator, experimental design, and manuscript preparation and revision. Both authors read and approved the final manuscript.

## Authors’ information

RC is the director of the Ponce Family and Youth Clinic of the Grady Infectious Disease Program serving the needs of over 400 HIV-infected children and adolescents within Atlanta Metropolitan Statistical Area and is the largest clinic of its kind in the US. RC serves as Chair of Committee on Pediatric Aids at the American Academy of Pediatrics. RC also serves as a member of the DHHS Panels on Treatment of HIV-Infected Pregnant Women and Prevention of Perinatal Transmission, and the Pediatric Antiretroviral Therapy and Management Guidelines. RC is also a member of the Perinatal HIV Advisory Council for the state of Georgia and also serves on a CDC task force panel to Eliminate Mother to Child Transmission in the US. Internationally, RC is a UK board member for an NGO in Nairobi, Kenya, which cares for HIV-infected orphans.

## Supplementary Material

Additional file 1 **HIV-1 replication in HCs.** HCs from multiple donors (N = 16) infected by HIV-1_BaL_*in vitro* showed differences in HIV-replication over time. HIV-1 replication was measured in the cell supernatants by HIV-1 p24 viral antigen ELISA. Data shown are expressed as the mean ± SE of triplicate samples. (PDF 148 kb) Click here for file

Additional file 2 **HIV-1 replication in MDMs.** MDMs from multiple donors (n = 8) infected by HIV-1_BaL_*in vitro* showed differences in HIV-replication over time. HIV-1 replication was measured in the cell supernatants by HIV-1 p24 viral antigen ELISA. Data shown are expressed as the mean ± SE of triplicate samples. (PDF 69 kb) Click here for file

Additional file 3 **EM of HIV-1 Infected HCs reveals Viral Assembly at the Plasma Membrane and within Intracellular Compartments.** HIV-1_Bal_ infected HCs were fixed and analyzed by standard electron microscope processing procedures. Sections show intracellular compartments with mature virions and immature assembly profiles (open arrows) (A). Virus assembly/budding profiles were also observed on the plasma membrane (PM) (B). Bars represent 0.2 μm for A and 0.1 μm for B. (PDF 131 kb) Click here for file
