# Supplementary figures and images for: Placental Hofbauer cells limit HIV-1 replication and potentially offset mother to child transmission (MTCT) by induction of immunoregulatory cytokines
Source: Retrovirology. 2012 Dec 5;9:101. doi: 10.1186/1742-4690-9-101 (PMC3524025; doi:10.1186/1742-4690-9-101)

Additional File 1

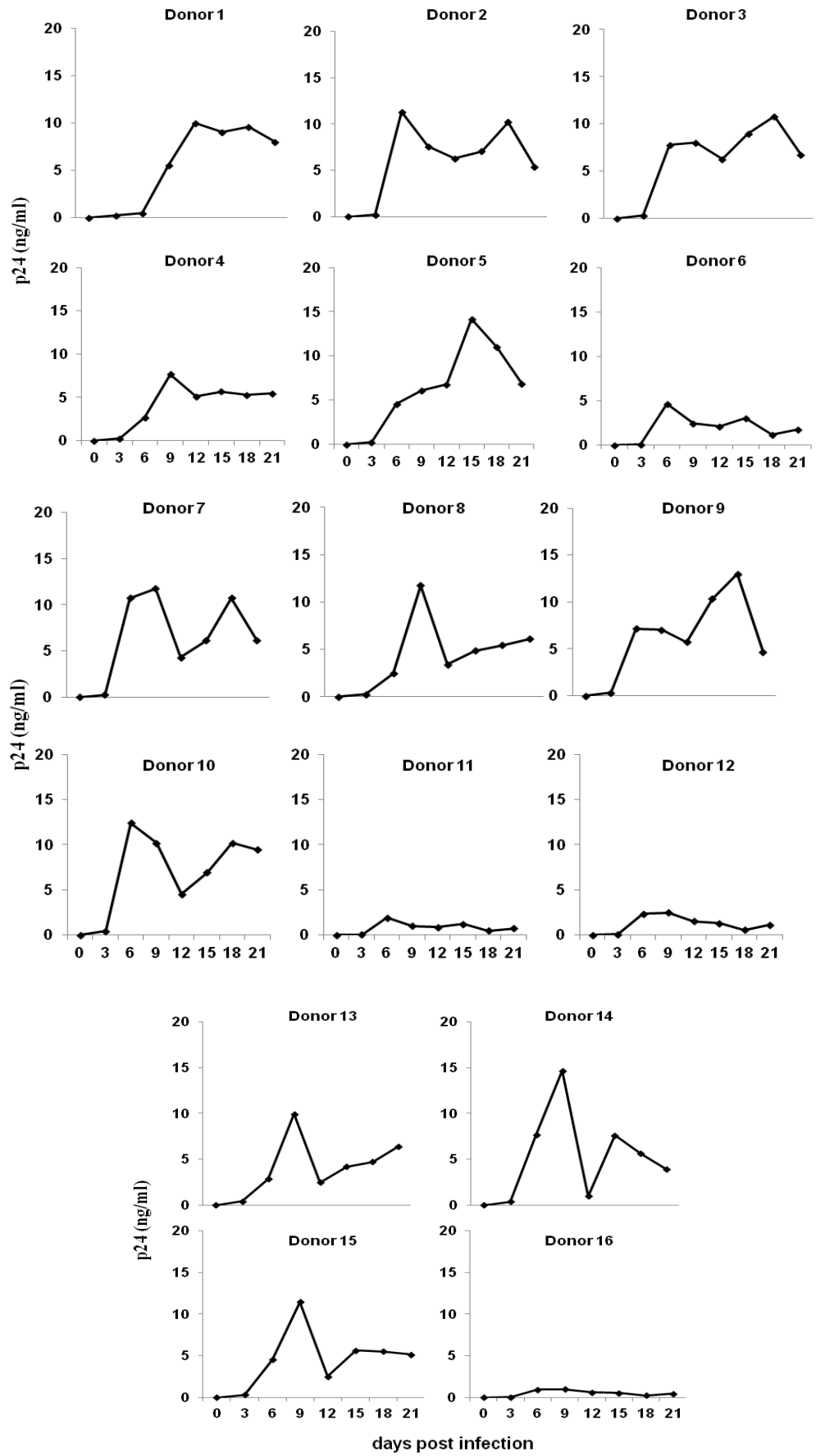

Supplement: Additional file 1 — HIV-1 replication in HCs. HCs from multiple donors (N = 16) infected by HIV-1BaLin vitro showed differences in HIV-replication over time. HIV-1 replication was measured in the cell supernatants by HIV-1 p24 viral antigen ELISA. Data shown are expressed as the mean ± SE of triplicate samples. (PDF 148 kb) [file 1742-4690-9-101-S1.pdf]

Additional File 2

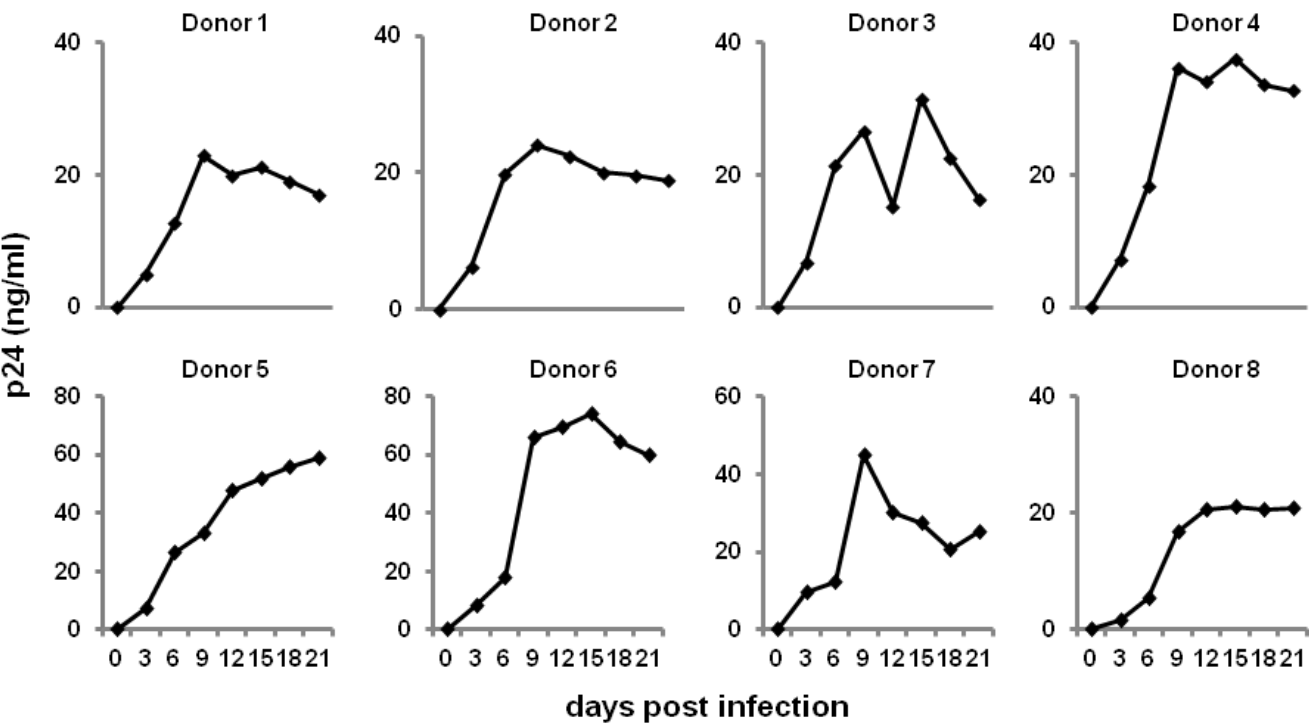

Supplement: Additional file 2 — HIV-1 replication in MDMs. MDMs from multiple donors (n = 8) infected by HIV-1BaLin vitro showed differences in HIV-replication over time. HIV-1 replication was measured in the cell supernatants by HIV-1 p24 viral antigen ELISA. Data shown are expressed as the mean ± SE of triplicate samples. (PDF 69 kb) [file 1742-4690-9-101-S2.pdf]

### Additional File 3

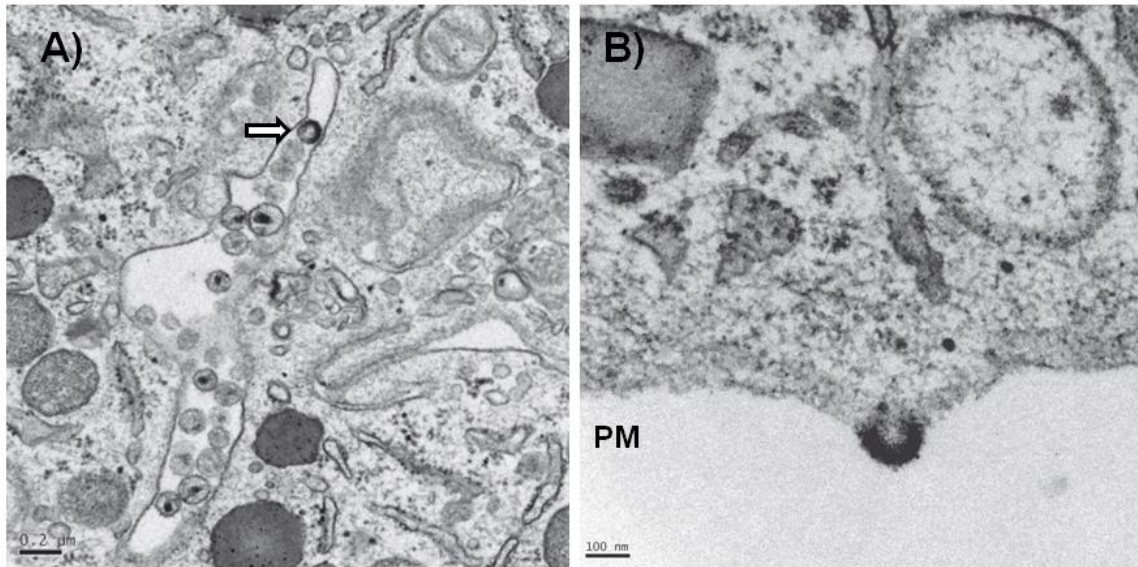

Supplement: Additional file 3 — EM of HIV-1 Infected HCs reveals Viral Assembly at the Plasma Membrane and within Intracellular Compartments. HIV-1Bal infected HCs were fixed and analyzed by standard electron microscope processing procedures. Sections show intracellular compartments with mature virions and immature assembly profiles (open arrows) (A). Virus assembly/budding profiles were also observed on the plasma membrane (PM) (B). Bars represent 0.2 μm for A and 0.1 μm for B. (PDF 131 kb) [file 1742-4690-9-101-S3.pdf]
